# Electrochemical Sensor for Methamphetamine Detection Using Laser-Induced Porous Graphene Electrode

**DOI:** 10.3390/nano12010073

**Published:** 2021-12-28

**Authors:** Kasrin Saisahas, Asamee Soleh, Sunita Somsiri, Patthamaporn Senglan, Kiattisak Promsuwan, Jenjira Saichanapan, Proespichaya Kanatharana, Panote Thavarungkul, Khai Lee, Kah Haw Chang, Ahmad Fahmi Lim Abdullah, Kunanunt Tayayuth, Warakorn Limbut

**Affiliations:** 1 Division of Health and Applied Sciences, Faculty of Science, Prince of Songkla University, Hat Yai, Songkhla 90110, Thailand; kasrin4223@gmail.com (K.S.); 6010210652@email.psu.ac.th (S.S.); 6010210158@email.psu.ac.th (P.S.); bejenji@gmail.com (J.S.); 2 Division of Physical Science, Faculty of Science, Prince of Songkla University, Hat Yai, Songkhla 90110, Thailand; asamee001@gmail.com (A.S.); promsuwan.k@gmail.com (K.P.); proespichk@gmail.com (P.K.); panote.t@psu.ac.th (P.T.); 3 Center of Excellence for Trace Analysis and Biosensors (TAB-CoE), Prince of Songkla University, Hat Yai, Songkhla 90110, Thailand; 4 Center of Excellence for Innovation in Chemistry, Faculty of Science, Prince of Songkla University, Hat Yai, Songkhla 90110, Thailand; 5 Forensic Science Programme, School of Health Sciences, Universiti Sains Malaysia, Kubang Kerian 16150, Malaysia; leekhai2@gmail.com (K.L.); changkahhaw@gmail.com (K.H.C.); fahmilim@usm.my (A.F.L.A.); 6 Science Park, Hat Yai Campus of Extension Southern Institute of Science Park, Prince of Songkla University, Moo 6, Thung Yai, Hat Yai, Songkhla 90110, Thailand; Kunanunt@gmail.com; 7 Forensic Science Innovation and Service Center, Prince of Songkla University, Hat Yai, Songkhla 90110, Thailand

**Keywords:** laser-induced porous graphene (LI-PGr), portable methamphetamine sensor, polyimide (PI), household surfaces, saliva sample

## Abstract

A 3D porous graphene structure was directly induced by CO_2_ laser from the surface of Kapton tape (carbon source) supported by polyethylene terephthalate (PET) laminating film. A highly flexible laser-induced porous graphene (LI-PGr) electrode was then fabricated via a facile one-step method without reagent and solvent in a procedure that required no stencil mask. The method makes pattern design easy, and production cost-effective and scalable. We investigated the performance of the LI-PGr electrode for the detection of methamphetamine (MA) on household surfaces and in biological fluids. The material properties and morphology of LI-PGr were analysed by scanning electron microscopy (SEM), energy dispersive x-ray (EDX) and Raman spectroscopy. The LI-PGr electrode was used as the detector in a portable electrochemical sensor, which exhibited a linear range from 1.00 to 30.0 µg mL^−1^ and a detection limit of 0.31 µg mL^−1^. Reproducibility was good (relative standard deviation of 2.50% at 10.0 µg mL^−1^; *n* = 10) and anti-interference was excellent. The sensor showed good precision and successfully determined MA on household surfaces and in saliva samples.

## 1. Introduction

Illegal drug use remains a global problem that threatens social stability, human health, and family harmony. The situation has worsened in recent years. Data released in the latest World Drug Report 2021 shows that over 275 million individuals used drugs worldwide over the past year, which is a 22 percent increase from 2010 [1]. Therefore, the development of rapid, sensitive, selective and cost-effective techniques for the in-situ identification of illicit drugs is necessary. One of the illegal drugs that most concerns the authorities is methamphetamine (MA), which is a highly addictive stimulant that profoundly affects the central nervous system [2]. MA can be produced on a small scale in any closed apartment for domestic use or on a bigger scale in clandestine laboratories with sophisticated production equipment [3]. The simple straightforward procedure of “cooking” MA has resulted in the establishment of clandestine laboratories in a number of locations and systems [4,5,6]. The identification of MA in biological and street samples is critical for organizations such as the Forensic Science Institute and the Office of Narcotics Control [7]. According to the Ministry of Public Health in Thailand, the lowest detectable concentration for the current MA test kit is 1 µg mL^−1^. 

In laboratories, MA analysis has involved techniques such as capillary electrophoresis, liquid chromatography-mass spectrometry (LC-MS), and gas chromatography-mass spectrometry (GC-MS) [8,9,10]. Despite the sensitivity and selectivity of these techniques, they may not be suitable for individual or on-site application due to the size and weight of the equipment, the need for skilled operators, and the high cost. In this context, electrochemical methods have great potential to meet this demand. Electrochemical systems can offer low-cost high performance and ease of use when coupled with handheld devices and mobile phone technology [11,12,13].

The development of electrochemical sensors has been promoted by the extensive use of carbonaceous nanostructured materials [14,15,16,17,18,19,20]. Graphene is known for its high surface area, rapid electron mobility, excellent conductivity, and mechanical stability [21,22,23,24,25]. Therefore, several methods of producing graphene with unique structures have evolved, including thermal decomposition [26,27], mechanical exfoliation [28,29], and the chemical vapor deposition and chemical/thermal reduction of graphite oxide [30,31,32]. Although the graphene products from these methods have perfectly suitable properties, the complicated procedures require a lot of chemicals, reagents, and time.

In 2014, Lin and co-workers reported a new, one-step approach that produced a graphene product known as laser-induced graphene (LIG). Using infrared (IR) laser irradiation technology, a polyimide (PI) substrate was modified into a porous graphene structure with a large specific surface area and excellent electrical conductivity [33]. LIG is created by photothermal reactions in which the sp^3^-carbon atoms on PI are converted into sp^2^-carbon atoms by IR laser irradiation. The energy from the laser irradiation creates lattice vibrations that cause high localized temperatures. The C-O, C=O, and N-C bonds in polyimide can be broken, recombined, and released as gases to produce a porous graphene nanostructure with pentagonal, heptagonal, and hexagonal lattice structures [33,34]. In addition, this fabrication technique has attracted considerable attention since it can produce graphitic structures from a variety of precursors without the usual high cost, flexible patterns, and chemicals [35,36,37]. Therefore, laser irradiation technology is an alternative approach to the production of 3D porous graphene electrodes for electrochemical sensors.

In this study, we present a simple, fast, inexpensive, and re-agentless strategy for the fabrication of a flexible, laser-induced porous graphene electrode (LI-PGr) using CO_2_ laser scribing on a PI precursor of Kapton tape reinforced with a thermal laminating PET film substrate. The optimized fabrication parameters of LI-PGr included laser speed and power, and the flexibility of the LI-PGr was tested. Three-dimensional printing technology was used to manufacture a portable electrochemical device that was integrated with a mobile phone application for convenient analysis in forensic investigation. The LI-PGr coupled with the developed portable electrochemical sensor was applied to investigate MA in saliva samples, as well as on nearby surfaces by means of surface recovery testing.

## 2. Materials and Methods

### 2.1. Chemicals and Apparatus

Acetic acid (CH_3_COOH), boric acid (H_3_BO_3_) and phosphoric acid (H_3_PO_4_) were purchased from Sigma-Aldrich (St. Louis, MI, USA). Sodium hydroxide was obtained from Merck (Darmstadt, Germany). Britton–Robinson (BR) buffer at pH 8.0 to 12.0 was prepared following a previously reported method [38]. All aqueous solutions were prepared with ultrapure water with a resistivity of 18 MΩ cm (Barnstead^TM^ EasyPure^TM^ II water purification system, Thermo Fischer Scientific^TM^, Waltham, MA, USA). Ag/AgCl ink (C2090225P7) was from Gwent Electronic Materials Co., Ltd. (Torfaen, UK). Laser patterning was carried out with a 50 W CO_2_ laser tube (XINGRUI (XR) laser, China). The Kapton tape (PI precursor, width 30 mm) and PET laminating film were purchased from local stores in Hat Yai, Thailand.

Surface morphologies were examined by scanning electron microscopy (SEM) (Quanta 400, FEI, Hillsboro, OR, USA) equipped with an Energy-Dispersive X-ray Spectroscopy (EDX) detector. EDX measurements (line spectra) were recorded using an operating voltage of 20 kV. Raman spectroscopy (Raman touch, Nanoproton, Japan) was performed using an excitation wavelength of 532.06 nm, a laser current of 100%, an excitation power of 0.44 mW, an excitation power density of 1.2 × 10^4^ W/cm^2^, an ND Filter of 0.13% (100/255), a wavenumber range from 0.00 to 4000.00 cm^−1^, a grating of 1200 gr/mm, and a slit width of 50 um. The signal was detected using a CCD detector. All of the experiments were carried out at ambient temperature (~25 °C).

### 2.2. Preparation of Laser-Induced Porous Graphene (LI-PGr) Electrode

**Figure 1:**
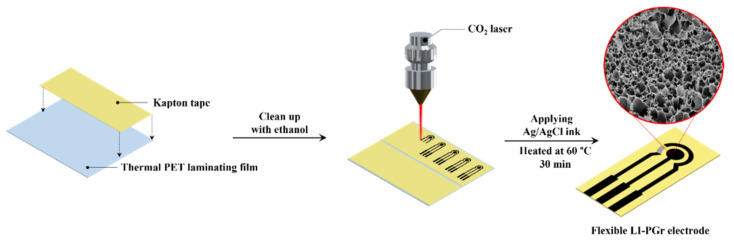
Schematic representation of the laser-induced porous graphene electrode fabrication.

A three-electrode system was designed with a working electrode (WE) of 3 mm in diameter, a counter electrode (CE), and a reference electrode (RE). The pattern was drawn with the aid of computer drawing software. To fabricate the electrode, Kapton tape was attached to thermal PET laminating film, and cleaned with ethanol to remove surface impurities. The electrode pattern was directly engraved in the Kapton tape with a CO_2_ laser operating at a power of 2.5 to 3.5% at a scan rate of 145–170 mm/sec. The RE was fabricated by painting Ag/AgCl ink onto the electrode with a paintbrush. The electrode was then dried for 30 min at 60 °C and, finally, individual LI-PGr electrodes were cut out with scissors. The procedure is illustrated in Figure 1.

### 2.3. Electrochemical Measurement and Characterization

**Figure 2:**
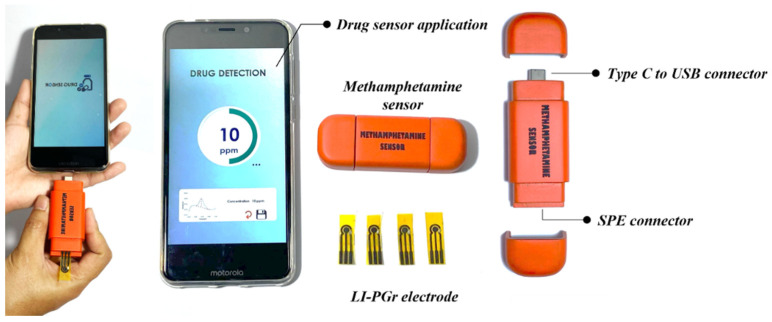
The components of the developed portable methamphetamine sensor.

All electrochemical experiments in this work used 30 µL sample solutions dropped onto the detection zone of the LI-PGr electrode. Electrochemical characterization, optimization, and analytical performance studies were enabled by a portable potentiostat (Emstat Pico, PalmSens, Houten, Netherlands) running the PSTrace program version 5.6. The electrochemical condition of cyclic voltammetric (CV) measurement with the fabricated electrode was optimized in BR buffer at pH 10.0, scanning the potential between +0.20 and +1.10 V at a rate of 50 mV s^−1^. Electrochemical impedance spectroscopy (EIS) parameters were set as follows: frequency ranged from 5.0 × 10^4^ Hz to 5.0 × 10^−2^ Hz; frequency number, 50; Eac, +0.01 V; Edc, +0.25 V. The parameters for differential pulse voltammetric (DPV) measurement were as follows: E pulse +0.25 V; t pulse 250 ms; E step +0.020 V with potential scanning between +0.20 V and +0.80 V at a rate of 40 mVs^−1^. Real samples were analysed using the developed portable methamphetamine sensor shown in Figure 2. The device consists of three major parts. There is the body of the device, which was manufactured by 3D printing. The body houses an Emstat Pico Module potentiostat, an SPE connector, and a USB-C connector. There is the sensing component, which is the LI-PGr electrode that connects to the device via the SPE connector. Lastly, there is the drug sensor application installed on the Android mobile phone, which connects to the device via a type C USB port. More details of the portable device are described in Supplementary Materials and Figure S1.

### 2.4. Sample Analysis

#### 2.4.1. Surface Recovery Experiment

The sample household materials were chosen from materials that were present during investigations of suspected MA cooking. They included glass, stainless steel, and plastic. MA samples were deposited by spraying 100 µL of a MA standard solution onto a 100 cm^2^ area outlined on the surface of the sample material. The concentrations of the solutions were 0.0, 50.0, and 100.0 µg mL^−1^, and when dried, the deposited traces of MA were of 0.0, 5.0, and 10.0 µg. Samples were collected by firmly wiping the area with filter paper saturated with methanol, a procedure adapted from Abdullah and Miskelly [39]. The surface wipe started in one corner of the square and followed a clockwise direction, then finished in the middle of the square, as shown in Figure S2. The filter was folded once more and the procedure was repeated, starting from another corner in an anticlockwise direction. The procedure was repeated 3 times. Once the area had been wiped, the filter paper containing the sample was placed in a 15 mL centrifuge tube. MA was extracted by sonication for 10 min in 5 mL of BR buffer added to the tube at pH 10.0. Finally, the filter paper was removed and 30 µL of extracted solution were dropped on the detection zone of the LI-PGr electrode for quantitative analysis.

#### 2.4.2. Saliva Sample

Saliva samples were collected from healthy individuals. Standard solutions of MA at 0.0, 5.0, 10.0, 15.0, 20.0, and 25.0 µg mL^−1^ were spiked into 1.0 mL sample aliquots.

## 3. Results

### 3.1. Optimization of LI-PGr Electrode Fabrication

**Figure 3:**
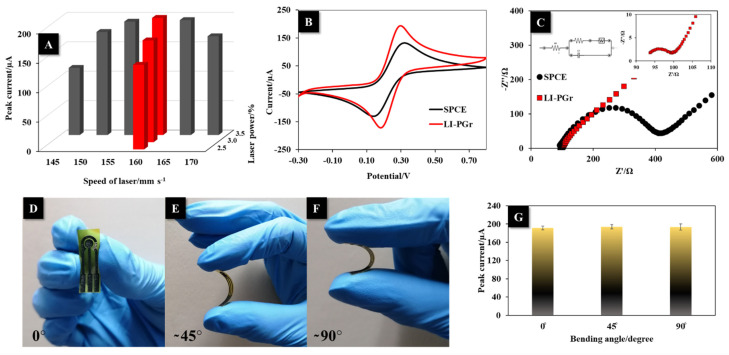
(**A**) The effects of laser speed and power on the fabrication of the laser-induced porous electrode were evaluated via the peak current response of 5.0 mM ferric/ferrocyanide ([Fe(CN)_6_]^3−/4−^). (**B**) CVs of a commercial SPCE and the LI-PGr electrode in ferric/ferrocyanide (5.0 mM) at 50 mV s^−1^. (**C**) EIS spectra of a commercial SPCE and the LI-PGr electrode in ferric/ferrocyanide (5.0 mM) were produced at frequencies between 5.0 × 10^4^ Hz and 5.0 × 10^−2^ Hz. Photographs are of the LI-PGr before bending (**D**) and after bending (**E**) at ~45° and (**F**) ~90°. (**G**) Effects of bending on the electrochemical performance of the LI-PGr electrode.

The laser power and speed used to fabricate the LI-PGr electrode were independently varied from 2.5 to 3.5% and from 145 to 170 mm s^−1^, respectively. The optimal parameters were those that produced the electrode that provided the highest peak current response of ferric/ferrocyanide ([Fe(CN)_6_]^3−/4−^) (5.0 mM) from CV measurement. The results indicated that the electrode that provided the highest peak current was produced using a laser power of 3.5% (1.75 W) and a laser speed of 160 mm s^−1^ (Figure 3A). Therefore, these conditions were chosen as the fabrication conditions of the LI-PGr electrode.

### 3.2. Electrochemical Characterization, Flexibility, and Stability Test of LI-PGr Electrode

The electrochemical properties of the LI-PGr electrode were compared to those of a commercial SPCE. The electrodes were applied to measure 5.0 mM [Fe(CN)_6_]^3−/4−^ using CV and EIS. The LI-PGr electrode produced a higher redox peak current and a smaller ΔE (E_p,a_-E_p,c_) of 120 mV as compared to the commercial SPCE (Figure 3B). The porous graphene fabricated by laser scribing had a larger surface area and excellent electrical conductivity compared to the flat commercial SPCE.

The EIS spectra of the LI-PGr electrode and commercial SPCE were produced in [Fe(CN)_6_]^3−/4−^ (5.0 mM). Nyquist diagrams (Figure 3C) displayed a typical semi-circular profile that indicated the occurrence of a charge-transfer-resistance-limiting process at high frequencies. The Nyquist plot data were then fitted to a Randles circuit (Figure 3C (inset)). The obtained charge-transfer resistance (Rct) values of the SPCE and LI-PGr electrode were found to be 260 ± 2 Ω and 4.7 ± 0.2 Ω, respectively. The smaller Rct of the LI-PGr demonstrated a higher electrical conductivity than the commercial SPCE. The charge-transfer rate constant (K_s_) values for both electrodes were calculated to determine the transfer rate of electrons between the electrode surface and target analyte. The K_s_ value can be estimated from the equation [40] K_s_ = RT/n^2^F^2^R_ct_C’, where K_s_, R_ct_, n, F, T, R, and C’, respectively, are the charge-transfer rate constant, the charge-transfer resistance, the number of electrons that are transferred, Faraday’s constant, the absolute temperature, the gas constant, and the concentration of the redox probe. The K_s_ values of the commercial SPCE and LI-PGr electrode obtained from the above equation were 2.04 × 10^−7^ and 1.13 × 10^−5^ m s^−1^, respectively. The obtained K_s_ values suggest that the porous structure of graphene induced by CO_2_ laser scribing could enhance electron transfer, which was 55.4 times higher between the target analyte and the LI-PGr electrode than between the target analyte and the commercial SPCE.

The LI-PGr electrode flexibility was tested by bending the electrodes at different angles for 1 min (Figure 3D–F) and comparing the electrochemical responses obtained by CV in [Fe(CN)_6_]^3−/4−^ (5.0 mM) before and after bending. The electrochemical responses of the LI-PGr electrode remained almost unchanged after bending at ~45° and ~90° (Figure 3G). These results indicate the excellent flexibility of the LI-PGr electrode platform.

The stability of the LI-PGr electrode was investigated by testing the lifetime of the electrode performance. Seven LI-PGr electrodes were constructed and stored in a locked plastic box filled with N_2_ gas and stored in a desiccator. The performance of the LI-PGr electrode was investigated after 2, 4, 6, 8, 10, 12, and 14 weeks by measuring [Fe(CN)_6_]^3−/4−^ (5.0 mM) using CV (Figure S3). It was found that, after 10 weeks, the LI-PGr electrode produced current responses of less than 80% of the initial response. The fabricated LI-PGr electrode could, therefore, be stored for up to 8 weeks without significant loss of performance.

### 3.3. Physical Characterizations

**Figure 4:**
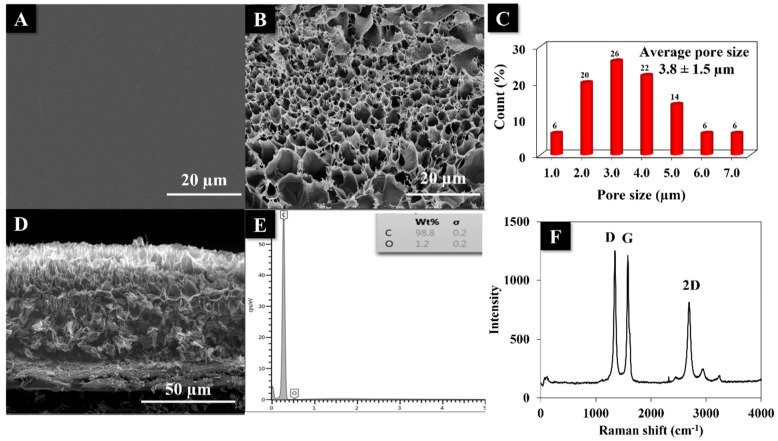
SEM images show Kapton tape before (**A**) and after (**B**) the laser scribing process. (**C**) An average pore size of the porous graphene structure. (**D**) Cross-sectional view of the LI-PGr electrode. (**E**) EDX spectrum and (**F**) Raman spectrum of the LI-PGr electrode.

The surface morphology of the LI-PGr electrode before and after laser scribing was recorded by SEM. The SEM image of Kapton tape before laser scribing (Figure 4A) displays a smooth surface. The SEM top-view shows a scribed region on the Kapton tape (Figure 4B). The 3D porous graphene structure was composed of interconnected fibrous strands with an average pore size of approximately 3.8 ± 1.5 µm (Figure 4C). The cross-sectional image (Figure 4D) is of LI-PGr induced by the CO_2_ laser at the optimal power and speed. A 3D porous graphene layer of around 61 ± 3 µm thickness was revealed. These porous structures enhanced the penetration of electrolytes to the active-surface sites of the LI-PGr electrode. The component elements of the LI-PGr were evaluated by EDX analysis. The EDX spectrum of the LI-PGr electrode (Figure 4E) revealed a composition of about 98.8% carbon and 1.2% oxygen.

The formation of graphene after the laser-inducing process was substantiated by Raman spectroscopy. The Raman spectrum of the LI-PGr electrode (Figure 4F) displays three notable peaks, including the D band, the G band, and a 2D band at approximately 1349, 1582, and 2690 cm^−1^, respectively. The G band was linked to vibrations in the plane of carbon bonds with sp^2^ hybridization. In contrast, the D and 2D bands demonstrate the primary in-plane and the second order in-plane vibrations, respectively. Additionally, the ratio of intensities between the D- and G-peaks (I_D_/I_G_) was used to evaluate the correlation of the degree of disorder of the carbon structure. The I_D_/I_G_ ratio of the fabricated LI-PGr electrode was 1.03, confirming the formation of graphene on the Kapton tape after laser scribing [41,42].

### 3.4. Electrochemical Behavior of MA on LI-PGr Electrode

**Figure 5:**
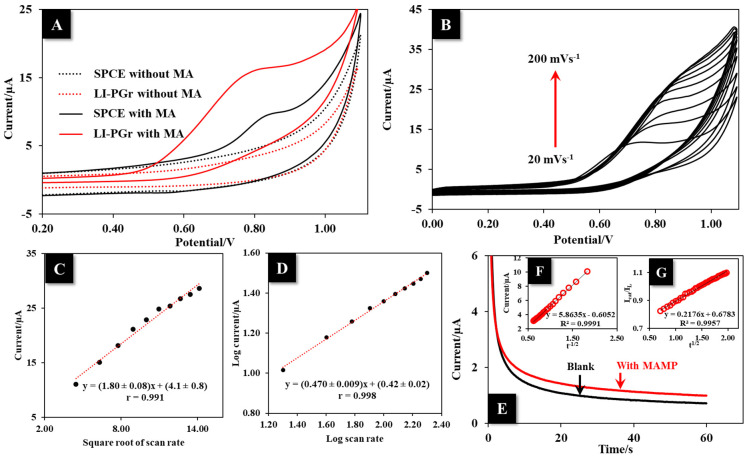
(**A**) CVs were obtained from an SPCE (black line) and the LI-PGr (red line) in BR buffer of pH 10.00 with (solid line) and without (dot line) 10 μg mL^−1^ MA. (**B**) CV responses at different scan rates (20–200 mV s^−1^) were produced at the LI-PGr electrode in the presence of 10.0 µg mL^−1^ MA. (**C**) The plot is of the square root of the scan rate (ν^1/2^) vs. the peak current (I). (**D**) The plot of log ν vs. log I. (**E**) i–t curves of LI-PGr electrode with and without 10.0 µg mL^−1^ MA at 0.70 V. (**F**) The plot of I vs. t^−1/2^ and (**G**) the plot of I_cat_/I_L_ vs. t^1/2^.

The electrochemical oxidation of MA at the commercial SPCE and LI-PGr electrode was evaluated using CV at a scan rate of 50 mV s^−1^ in BR buffer (pH 10.0) with and without 10.0 μg mL^−1^ MA. Without MA, only the background current was observed for both the LI-PGr (red dot line) and the commercial SPCE (black dot line) electrodes (Figure 5A). Notably, the LI-PGr electrode showed a background current smaller than that of the commercial SPCE, implying that the LI-PGr electrode possesses excellent conductivity but produces a low capacitive current, which is more suitable for analytical purposes. The results obtained in the presence of MA revealed the irreversible behavior of the analyte at both electrodes, which produced only oxidation peaks. The anodic peak current at the LI-PGr was about 221% that of the commercial SPCE. These results show that the oxidation of MA at the LI-PGr electrode was excellent, which can be attributed to the high electroactive surface area and conductivity of the LI-PGr electrode.

To better understand the electron transport mechanism that occurs at the LI-PGr electrode surface, the influence of the scan rate on the oxidation peak of MA was investigated using CV. For 10.0 μg mL^−1^ of MA, the anodic peak current increased continuously with the scan rate from 20 to 200 mV s^−1^ (Figure 5B). As regards the results, increasing the scan rate shifted the oxidation peak potential to a more positive potential, which implied the influence of kinetics limitation in the electrochemical reaction. Figure 5C shows the linear plot of the peak current (I_p_) against the square root of the scan rate (ν^1/2^) following the linear regression equation of I_p_ = (1.80 ± 0.08) ν^1/2^ + (4.1 ± 0.8) with a correlation coefficient equal to 0.991. The plot indicated that MA oxidation at the LI-PGr electrode surface was a diffusion-controlled process. In addition, the relationship of log peak current versus log scan rate (log I_p_ vs. log ν ) was used to typify the kinetic behavior of the reaction. The plot in Figure 5D exhibits the linear relationship of log I_p_ vs. log ν, in which the linear equation was log I_p_ = (0.470 ± 0.009) log ν − (0.42 ± 0.02); r = 0.998. The obtained slope value of 0.470 ± 0.009 was close to the theoretical value of 0.5, which confirmed the reaction as a diffusion-controlled process.

The theoretical assumption of the diffusion-controlled behavior of MA at the LI-PGr electrode surface was tested by investigating the anodic peak current response after increasing the pre-concentration time of MA from 0 to 90 s. The anodic current responses of MA did not change significantly when the preconcentration time was increased (Figure S4). This result confirmed that the oxidation reaction of MA at the LI-PGr electrode surface was completely controlled by diffusion, and thus, there was no need to apply a preconcentration step to increase the signal in the determination of MA.

The MA diffusion coefficient (D) and catalytic rate constant (k_cat_) of the LI-PGr electrode toward MA oxidation were estimated using a chronoamperometric method by measuring 10.0 μg mL^−1^ MA in BR buffer at pH 10.0 at a potential of 0.70 V (Figure 5E). The D value of MA at the LI-PGr electrode was estimated by employing the slope value of the I vs. t^−1/2^ plot (5.8 µA s^1/2^) (Figure 5F) with the Cottrell equation, I = nFAD^1/2^C/π^1/2^t^1/2^ [43], where n, A, and C, respectively, are the electron number, the real surface area, and the concentration of the analyte. The D value of MA at the LI-PGr electrode was 1.21 × 10^−5^ cm^2^ s^−1^. The k_cat_ value of the LI-PGR toward MA oxidation was estimated from the slope of the I_Cat_/I_L_ vs. t^1/2^ plot (0.22 s^−1/2^) (Figure 5G) using the Galus equation, I_Cat_/I_L_ = π ^1/2^(k_cat_C_b_t)^1/2^ [44], where I_Cat_, I_L_, C, and t, respectively, are the current responses of MA and the blank, the concentration of MA, and time. The k_cat_ value was calculated to be 2.26 × 10^5^ mol^−1^ L s^−1^.

### 3.5. Effect of pH

The pH of the BR buffer was an essential factor that affected the electrochemical response of MA on the LI-PGr electrode. The effect was evaluated using DPV in 0.04 M BR buffer at pH levels ranging from 8.0 to 12.0 (Figure S5). The peak current gradually increased with increments of pH from 8.0 to 10.0 and decreased with increments from 10.0 to 12.0. Since MA oxidation is related to the donation of lone-pair electrons at the secondary amino group of MA, this phenomenon can be described using the pKa value of MA, which is 9.87. The protonation of MA can occur at pH levels of between 8.0 and 9.0, in which case the MA molecule is oxidized with difficulty. In contrast, at pH levels above 10.0, the deprotonation of the secondary amino group of the MA molecule can occur. Therefore, BR buffer at pH 10.0 was selected for MA detection.

### 3.6. MA Detection using the LI-PGr Electrode

**Figure 6:**
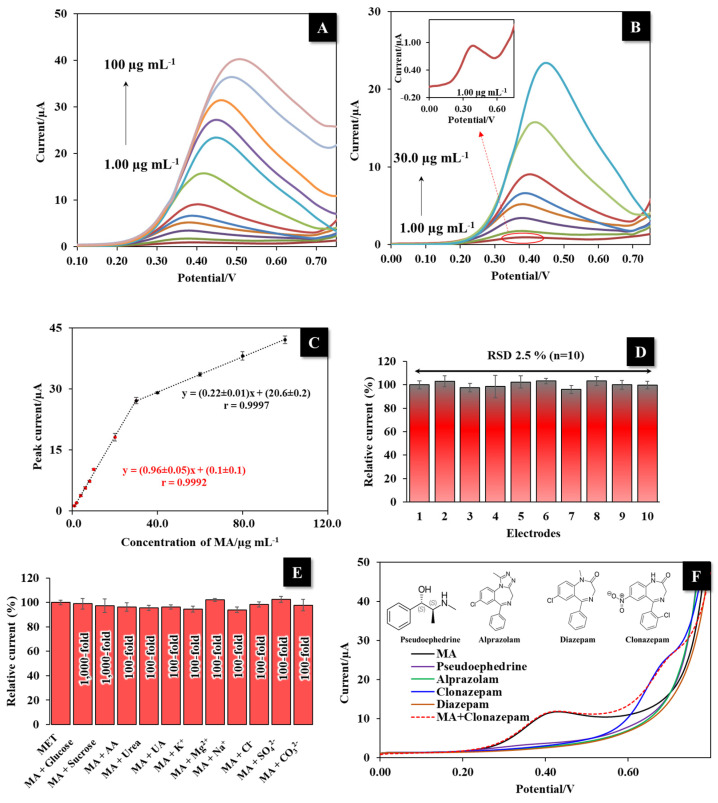
(**A**) DPV responses of MA (1.00 to 100 µg mL^−1^) at the LI-PGr electrode coupled with the developed portable device. (**B**) DPV responses of MA (1.00 to 30 µg mL^−1^) at the LI-PGr electrode; inset—amplified anodic peak current of MA at a concentration of 1.00 µg mL^−1^. (**C**) Calibration curve of current signal of MA versus its concentration (1.00–100 µg mL^−1^). (**D**) The relative current responses from ten LI-PGr electrode preparations. (**E**) Anti-interference ability of the developed sensor. (**F**) DPV responses of MA (10.0 µg mL^−1^), pseudoephedrine (10.0 µg mL^−1^), alprazolam (10.0 µg mL^−1^), clonazepam (10.0 µg mL^−1^), diazepam (10.0 µg mL^−1^), and a mixed solution of MA (10.0 µg mL^−1^) and clonazepam (10.0 µg mL^−1^).

The analytical performance of the LI-PGr electrode for the MA detection was investigated using the DPV technique due to its low charging contribution to background current and high current response. The anodic peak current of MA at concentrations between 1.00 and 100 µg mL^−1^ as shown in Figure 6A.

In addition, Figure 6B shows the amplified anodic peak current of MA at concentrations of between 1.00 and 30.0 µg mL^−1^ and the inset of Figure 6B shows the amplified anodic peak current of MA at a concentration of 1.00 µg mL^−1^. The maximum anodic peak of MA occurred at +0.40 V, and linearly increased with the MA concentration. The LI-PGr electrode-based sensor presented two linear ranges of MA detection: from 1.00 to 30.0 µg mL^−1^ and from 30.0 to 100 µg mL^−1^ (Figure 6C). The DPV response in this study exhibited a shift in response potential due to the material-dependent rate of current transfer from the bulk solution to the electrode, which is termed “mass transport”. Diffusion, where molecules in a high-concentration region randomly move to a low-concentration region, occurs satisfactorily when the analyte is at a low concentration. However, at high concentrations, the diffusion process is disrupted. In this case, mass transport is consequently affected. Hence, to compensate and re-establish mass transport, the increased potential is applied by the electrochemical system, subsequently leading to a shift in the peak potential [45,46]. The limit of detection (LOD) was 0.31 µg mL^−1^ (LOD = 3*S.D. of the intercept/slope of the calibration curve). The analytical performances obtained with our portable MA sensor were compared with the performances of other electrochemical sensors for the detection of MA (Table S1). The proposed sensor did not return the lowest LOD or widest linear range when compared with other electrochemical sensors. However, this proposed sensor exhibited ease of preparation and could prepare three electrodes on one single piece using a one-step laser irradiation system. Moreover, only 30 µL of sample solution was used for electrochemical measurement and this sensor provided a convenient method of detecting MA with a portable device that produced highly accurate results from a rapid, simple, and stable electrode fabrication process. In addition, the proposed sensor developed in this study had sufficient sensitivity to detect the concentration of methamphetamine according to the requirements of the Ministry of Public Health in Thailand; the lowest detectable concentration for the current methamphetamine test kit is 1 µg mL^−1^.

### 3.7. Reproducibility and Interference Study

The reproducibility of the LI-PGr electrode was assessed by evaluating the current signal from ten electrodes prepared in the same condition. Figure 6D shows the relative current response of 10.0 µg mL^−1^ MA from ten electrode repetitions. The RSD was 2.50%, which is acceptable according to the Association of Analytical Communities (AOAC) guidelines [47]. This result indicates the good reproducibility of the LI-PGr electrode preparation and MA detection.

The effects of interferences on MA detection with the developed LI-PGr electrode were tested by measuring 10.0 µg mL^−1^ MA in the presence of the common interferences, glucose, sucrose, ascorbic acid, urea, uric acid, K^+^, Mg^2+^, Na^+^, Cl^−^, SO_4_^2−^, and CO_3_^2−^. No interference was observed in the presence of 1000-fold concentrations of glucose and sucrose, and 100-fold concentrations of urea, uric acid, ascorbic acid, Na^+^, Mg^2+^, K^+^, Cl^−^, SO_4_^2−^, and CO_3_^2−^ (Figure 6E). Furthermore, the selectivity of the LI-PGr electrode was also tested by comparing it to some other compounds such as pseudoephedrine, alprazolam, clonazepam, and diazepam. The results shown in Figure 6F demonstrate that pseudoephedrine, alprazolam, clonazepam, and diazepam have no significant current signal (despite pseudoephedrine having high structural similarity to MA) using the LI-PGr sensor, with the exception of clonazepam. However, in a mixture of MA and clonazepam, with the same concentration of both, the obtained current signal for MA and clonazepam could clearly separate the two with no significant current signal and potential change. As a result of this finding, the LI-PGr electrode appears to be highly selective for MA.

### 3.8. Application with Real Samples

The applicability of the proposed LI-PGr electrode and a developed portable MA sensor device was evaluated by detecting MA in two types of samples: household surfaces and biological fluids (i.e., saliva). In this study, trace amounts of MA were recovered from common household surfaces including glass, stainless steel, and plastic. MA was deposited at 0, 5, and 10 µg/100 cm^2^ onto selected surfaces and measured with the developed portable device coupled with the LI-PGr electrode. Figure S6 shows the DPV response of MA on each surface sample. The obtained percent recoveries were between 84.1 ± 0.9 and 98.4 ± 0.3 (*n* = 3) (Table 1). In addition, the measurement of MA in the saliva sample was demonstrated by determining MA standards spiked at 5, 10, 15, 20, and 25 μg mL^−1^. The percent recoveries obtained ranged from 84 ± 4 to 104 ± 8 (*n* = 3) (Table 2). These good recovery results indicated that the new, simple electrochemical sensor platform proposed here for MA detection could successfully be applied in forensic investigation.

## 4. Conclusions

In summary, we created a new, simple, and extremely flexible laser-induced porous graphene electrode as well as a portable electrochemical device for methamphetamine screening and quantification. CO_2_ laser scribing on a polyimide precursor of Kapton tape, on a substrate of polyethylene terephthalate thermal laminating film, was used to create the laser-induced porous graphene electrode in a quick procedure. The housing of a portable electrochemical drug sensor was made using a 3D printing system, and it was connected to a mobile phone running an interface application. The proposed electrode was sensitive and specific to methamphetamine and had a highly porous graphene structure with excellent conductivity. Furthermore, the sensor was used to detect methamphetamine on household surfaces as well as in saliva samples. It was possible to conduct recovery on these surfaces and samples in a satisfactory manner. Finally, we believe that the developed portable sensor device, in combination with the fabricated porous graphene electrode, has great potential in forensic investigation and other fields.

## Figures and Tables

**Table 1:** Recoveries of MA on household surfaces including glass, stainless steel, and plastic from surface areas of 100 cm^2^.

Common Household Surface	Spiked (µg/100 cm^3^)	Found (µg mL^−1^) (*n* = 3)	% Recovery (*n* = 3)
Glass	0	N.D.	-
	5	5.6 ± 1.2	98.4 ± 0.3
	10	9.0 ± 0.5	90 ± 5
Stainless steel	0	N.D.	-
	5	4.3 ± 0.3	86 ± 6
	10	8.41 ± 0.09	84.1 ± 0.9
Plastic	0	N.D.	-
	5	4.4 ± 0.2	87 ± 3
	10	8.6 ± 0.3	86 ± 3

N.D.: not detected.

**Table 2:** Determination of MA concentrations in saliva sample using the proposed LI-PGr electrode coupled with a developed portable device with the recovery values of MA from saliva sample.

Saliva Sample	Spiked (µg mL^−1^)	Found (µg mL^−1^) (*n* = 3)	% Recovery (*n* = 3)
S1	0	N.D.	-
S2	5	4.2 ± 0.2	84 ± 4
S3	10	10.4 ± 0.5	104 ± 5
S4	15	16 ± 2	104 ± 8
S5	20	19.9 ± 0.9	99 ± 4
S6	25	24.3 ± 0.7	97 ± 3

N.D.: not detected.

## Data Availability

The data presented in this study are available on request from the corresponding author.

## Supplementary Materials

The following are available online at https://www.mdpi.com/article/10.3390/nano12010073/s1, Figure S1: A portable electrochemical device and fully functional EmStat Pico USB connection with a USB to UART convertor to interface with a type-C USB connection and an SPE connector, Figure S2: Illustration of wiping pattern on a sampling area of 100 cm^2^ during the surface recovery experiment, Figure S3: CVs and relative current response of 5.0 mM ferric/ferrocyanide on the LI-PGr electrode at different storage times (2, 4, 6, 8, 10, 12, and 14 weeks), Figure S4: The effect of different pre-concentration times (0, 30, 60, and 90 s) on the peak current of 10.0 µg mL^−1^ MA at the LI-PGr electrode, Figure S5: The effect of pH buffer on the peak current of 10.0 µg mL^−1^ MA at the LI-PGr electrode, Figure S6: DPV responses of MA on the glass surface at the concentration 0.0 (A), 5.0 (B) and 10.0 µg mL^−1^ (C); on the stainless-steel surface at the concentration 0.0 (D), 5.0 (E) and 10.0 µg mL^−1^ (F); and on the plastic surface at the concentration 0.0 (G), 5.0 (H) and 10.0 µg mL^−1^ (I). Table S1: Comparison of analytical performances of the proposed MA sensor with some previously reported MA sensors.
