# Supplementary material for: Electrochemical Sensor for Methamphetamine Detection Using Laser-Induced Porous Graphene Electrode
Source: Nanomaterials (Basel). 2021 Dec 28;12(1):73. doi: 10.3390/nano12010073 (PMC8746692; doi:10.3390/nano12010073)
Supplement: Supplementary file 1 [file nanomaterials-12-00073-s001.zip › nanomaterials-1485432-supplementary.pdf]

nanomaterials

*Supplementary Materials*

# Electrochemical Sensor for Methamphetamine Detection using Laser-induced Porous Graphene Electrode

**Kasrin Saisahas [1], Asamee Soleh [2,3,4], Sunita Somsiri [1], Patthamaporn Senglan [1], Kiattisak Promsuwan [2,3,4], Jenjira Saichanapan [1], Proespichaya Kanatharana [2,3,4], Panote Thavarungkul [2,3,4], Khai Lee [5], Kah Haw Chang [5], Ahmad Fahmi Lim Abdullah [5], Kunanunt Tayayuth [6], Warakorn Limbut [1,3,4,7*]**

[1] Division of Health and Applied Sciences, Faculty of Science, Prince of Songkla University, Hat Yai, Songkhla 90110, Thailand; kasrin4223@gmail.com (K.S.); 6010210652@email.psu.ac.th (S.S.); 6010210158@email.psu.ac.th (P.S.); bejenji@gmail.com (J.S.)
[2] Division of Physical Science, Faculty of Science, Prince of Songkla University, Hat Yai, Songkhla 90110, Thailand; asamee001@gmail.com (A.S.); promsuwan.k@gmail.com (K.P.); proespichk@gmail.com (P.K.); panote.t@psu.ac.th (P.T.)
[3] Center of Excellence for Trace Analysis and Biosensors (TAB-CoE), Prince of Songkla University, Hat Yai, Songkhla 90110, Thailand
[4] Center of Excellence for Innovation in Chemistry, Faculty of Science, Prince of Songkla University, Hat Yai, Songkhla 90110, Thailand
[5] Forensic Science Programme, School of Health Sciences, Universiti Sains Malaysia, Kubang Kerian 16150, Kelantan, Malaysia; leekhai2@gmail.com (K.L.); changkahhaw@gmail.com (K.H.C.); fahmilim@usm.my (A.F.L.A.)
[6] Science Park, Hat Yai Campus of Extension Southern Institute of Science Park, Prince of Songkla University, Moo 6, Thung Yai, Hat Yai, Songkhla 90110, Thailand; Kunanunt@gmail.com
[7] Forensic Science Innovation and Service Center, Prince of Songkla University, Hat Yai, Songkhla 90110, Thailand
* Correspondence: warakorn.l@psu.ac.th; Tel.: +66-74-288-563

***Portable electrochemical device fabrication***

The portable electrochemical device contains an Emstat Pico Module potentiostat (PalmSens, www.palmsens.com/product/oem-emstat-pico-module/). The Emstat Pico Module potentiostat will connected to two connectors, including a USB to UART convertor (UMFT234XD-NC) used for connecting to a smartphone via a type-C USB connector and a screen-printed electrode (SPE) connector (DS1020-03ST1D) used for connecting to the LIG electrode (Figure S1). The body of the device was designed a three-dimensional (3D) model using the program Solid work 2020 and created with 3D Printer (GEEETECH E180 3D printer) by the fused deposition modeling (FDM) method using poly (lactic acid) (PLA) filament.

For the android smartphone (Motorola One) running the drug sensor application was used to control the portable electrochemical device. This application was developed from PalmSens Software Development Kits (SDKs) for .NET (www.palmsens.com/oem/sdkdotnet/), which has two modes (i) standard curve for standard detection and (ii) drug detection for real-sample analysis.

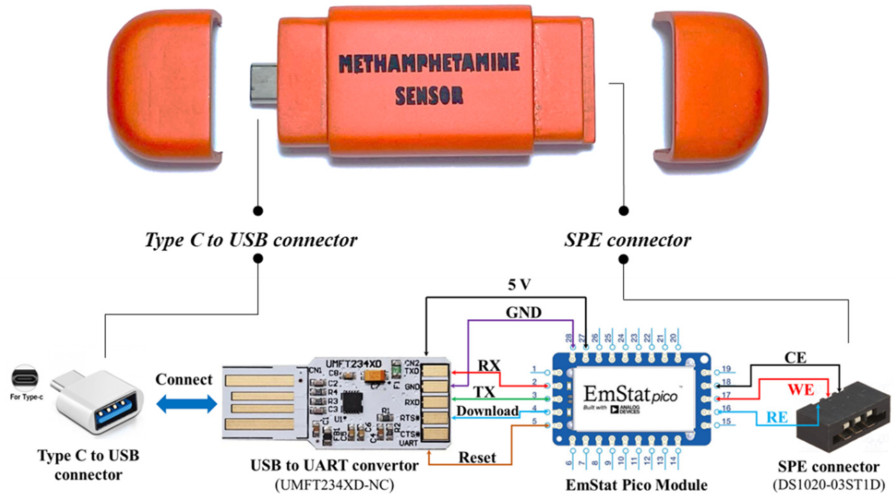


**Figure S1.** A portable electrochemical device and fully functional EmStat Pico USB connection with a USB to UART convertor to interface with a type-C USB connection and an SPE connector.

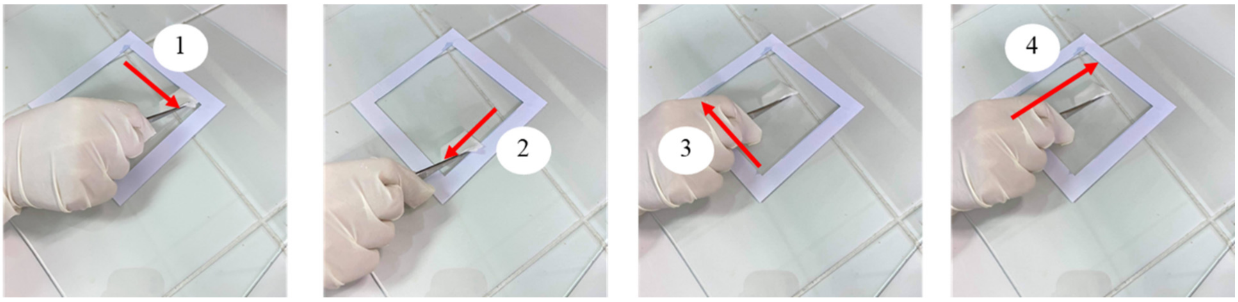


**Figure S2.** Illustration of wiping pattern on a sampling area of 100 $cm^2$ during the surface recovery experiment.

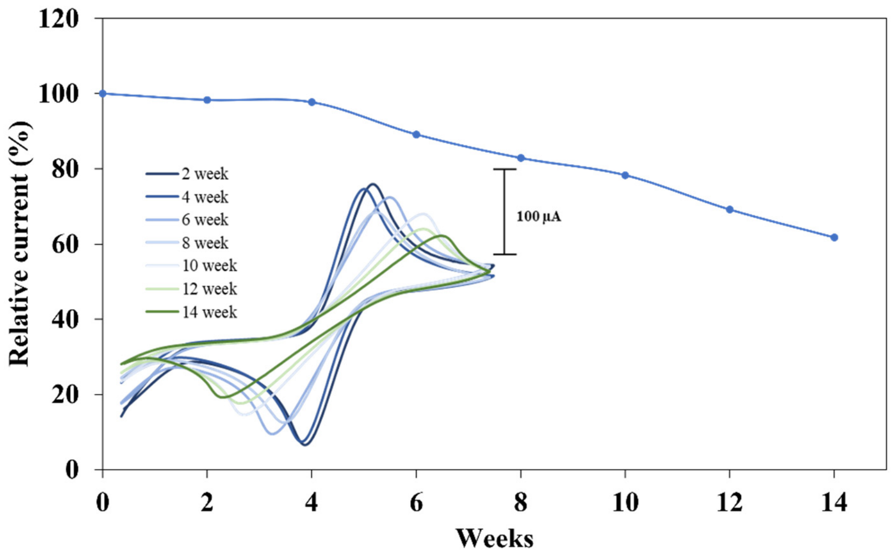


**Figure S3.** CVs and relative current response of 5.0 mM ferric/ferrocyanide on the LI-PGr electrode at different storage times (2, 4, 6, 8, 10, 12, and 14 weeks).

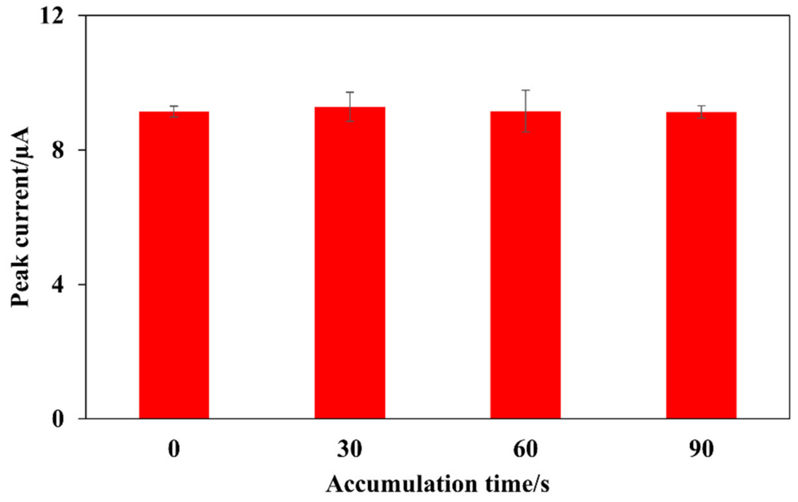


**Figure S4.** The effect of different pre-concentration times (0, 30, 60, and 90 s) on the peak current of 10.0 μg mL$^{-1}$ MA at the LI-PGr electrode.

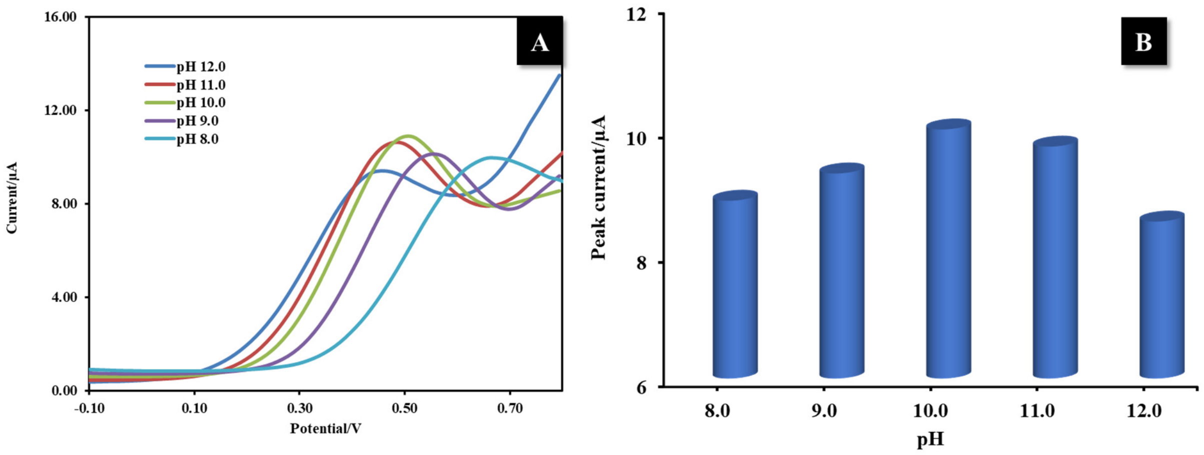


**Figure S5.** The effect of pH buffer on the peak current of 10.0 μg mL$^{-1}$ MA at the LI-PGr electrode.

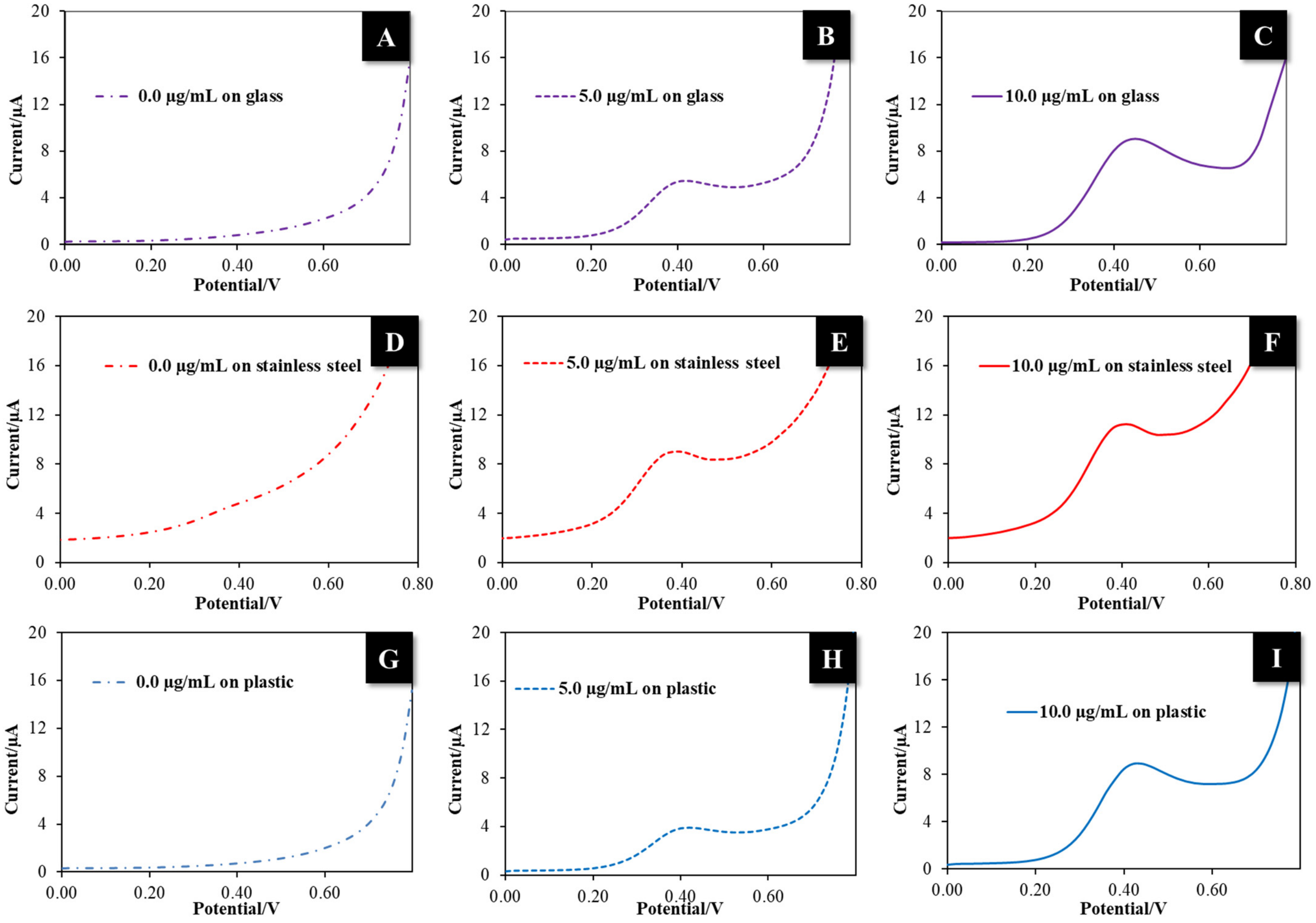


**Figure S6.** DPV responses of MA on the glass surface at the concentration of 0.0 (A), 5.0 (B) and 10.0 µg mL$^{-1}$ (C); on the stainless-steel surface at the concentration of 0.0 (D), 5.0 (E) and 10.0 µg mL$^{-1}$ (F); and on the plastic surface at the concentration of 0.0 (G), 5.0 (H) and 10.0 µg mL$^{-1}$ (I).

**Table S1.** Comparison of analytical performances of the proposed MA sensor with some previously reported MA sensors.

| Electrode | Method | Portability | Linear range (µg mL$^{-1}$) | Limit of detection (µg mL$^{-1}$) | Ref. |
|---|---|---|---|---|---|
| LI-PGr electrode | DPV | Yes | 1.00 – 30.0, 30.0 - 100 | 0.31 | This work |
| MIP/MWCNTs/CPE | FFT-SWV | No | 0.0015 – 15 | 0.00012 | [1] |
| MWCNT/Au-NPsHS-$SiO_2$/$Fe_3O_4$ | SWV | No | 0.0075 – 7.5 | 0.0024 | [2] |
| PPGE | DPV | No | 0.01 – 8.0 | 0.0075 | [3] |
| GE/LC/PB/MPS/AuNP/Anti - MA | Amp | No | 0.015 – 0.75 | 0.001 | [4] |
| Aptamer/AuNPs/Chitosan/GCE | CV | No | - | 0.0015 | [5] |
| GCE/EDOT-BTDA-Pala/Antibody/ MA | DPV | No | 10-100 | 0.014 | [6] |

## References


1. Akhoundian, M.; Alizadeh, T.; Ganjali, M.R.; Norouzi, P. Ultra-trace detection of methamphetamine in biological samples using FFT-square wave voltammetry and nano-sized imprinted polymer/MWCNTs -modified electrode. *Talanta* **2019**, *200*, 115-123, doi:https://doi.org/10.1016/j.talanta.2019.02.027.
2. Haghighi, M.; Shahlaei, M.; Irandoust, M.; Hassanpour, A. New and sensitive sensor for voltammetry determination of Methamphetamine in biological samples. *Journal of Materials Science: Materials in Electronics* **2020**, *31*, 10989-11000, doi:10.1007/s10854-020-03647-6.
3. Oghli, A.H.; Alipour, E.; Asadzadeh, M. Development of a novel voltammetric sensor for the determination of methamphetamine in biological samples on the pretreated pencil graphite electrode. *RSC Advances* **2015**, *5*, 9674-9682, doi:10.1039/C4RA11399C.
4. Zhang, L.-Y.; Liu, Y.-J. Label-free amperometric immunosensor based on prussian blue as artificial peroxidase for the detection of methamphetamine. *Analytica Chimica Acta* **2014**, *806*, 204-209, doi:https://doi.org/10.1016/j.aca.2013.11.019.
5. Kohzadi, R.; Molaeirad, A.; Alijanianzadeh, M.; Kamali, N.; Mohtashamifar, M. Designing a Label Free Aptasensor for Detection of Methamphetamine. *Biomacromolecular Journal* **2016**, *2*, 28-33.
6. Demir, B.; Yilmaz, T.; Guler, E.; Gumus, Z.P.; Akbulut, H.; Aldemir, E.; Coskunol, H.; Colak, D.G.; Cianga, I.; Yamada, S.; et al. Polypeptide with electroactive endgroups as sensing platform for the abused drug ‘methamphetamine’ by bioelectrochemical method. *Talanta* **2016**, *161*, 789-796, doi:https://doi.org/10.1016/j.talanta.2016.09.042.
